# Effect of Ce Addition on Microstructure, Thermal Conductivity, and Mechanical Properties of As-Cast and As-Extruded Mg–3Sn Alloys

**DOI:** 10.3390/ma17174251

**Published:** 2024-08-28

**Authors:** Fei-yu He, Wen-xin Hu, Li-juan Liu, Wei He, Shao-bo Ma, Xu-dong Zhang, Zheng-hua Yang, Wei Wang

**Affiliations:** 1 National Key Laboratory of Baiyunobo Rare Earth Resource Researches and Comprehensive Utilization, Baotou 014030, China; hefeiyu@brire.com (F.-y.H.); he13030462937@126.com (W.H.); shaonoma@brire.com (S.-b.M.); xudongzhang@brire.com (X.-d.Z.); yangzhenghua@brire.com (Z.-h.Y.); 13664774273@163.com (W.W.); 2 Baotou Research Institute of Rare Earths, Baotou 014030, China; 3 Technical Center of Steel Union Co., Ltd. of Baotou Steel (Group) Corp, Baotou 014010, China; bggf_jishuzhongxin@163.com

**Keywords:** Mg-3Sn alloy, thermal conductivity, mechanical properties, microstructure, Ce addition

## Abstract

In the present research, the impacts of Ce additions at various concentrations (0, 1.0, 3.4, and 4.0 wt.%) on the evolution of the microstructure, mechanical properties, and thermal conductivity of as-cast and as-extruded Mg-3Sn alloys were investigated. The findings demonstrate that adding Ce caused the creation of a new ternary MgSnCe phase in the magnesium matrix. Some new Mg_17_Ce_2_ phases are generated in the microstructure when Ce levels reach 4%. The thermal conductivity of the Mg-3Sn alloy is significantly improved due to Ce addition, and the Mg-3Sn-3.4Ce alloy exhibits the highest thermal conductivity, up to 133.8 W/(m·K) at 298 K. After extrusion, both the thermal conductivity and mechanical properties are further improved. The thermal conductivity perpendicular to the extrusion direction of Mg-3Sn-3.4Ce alloy could achieve 136.28 W/(m·K), and the tensile and yield strengths reach 264.3 MPa and 227.2 MPa, with an elongation of 7.9%. Adding Ce decreases the dissolved Sn atoms and breaks the eutectic *α*-Mg and Mg_2_Sn network organization, leading to a considerable increase in the thermal conductivity of as-cast Mg-3Sn alloys. Weakening the deformed grain texture contributed to the further enhancement of the thermal conductivity after extrusion.

## 1. Introduction

Magnesium alloys are lightweight structural materials with low density, high strength, and excellent vibration damping properties. It is commonly applied in automobiles, aircraft, and spacecraft fields [1,2,3]. The functional properties of magnesium alloys are increasingly being emphasized alongside their mechanical properties. In particular, thermal conductivity is one of the major focuses of new electronic products (LEDs, computers, etc.) today [4,5,6]. However, there is an imbalance between the development of thermal conductivity and the mechanical properties of existing magnesium alloys in general, like the commonly used standard magnesium alloy AZ91D with a room temperature yield strength of around 160 MPa and a thermal conductivity of only 51 W/(m·K) [7]. Therefore, the key to expanding the engineering applications of magnesium alloys is to balance the mechanical properties and thermal conductivity.

Mg-Sn alloys have aroused concerns owing to their excellent mechanical properties and high creep properties at elevated temperatures [8]. In recent decades, a great deal of work has been done to enhance the mechanical properties of Mg-Sn-based alloys by adding other elements [9]. Most have been extensively developed by adding components like Zn, Mn, Y, Al, and Ca. After adding Zn to a Mg-3Sn-1Al alloy, Chen [10] found that with the increases in Zn content, the grain size had been improved and the size of the fine Mg_2_Sn particles significantly increased. Pan [11] et al. discovered that the composite addition of Zn and Ca can effectively improve the mechanical properties of Mg-2Sn alloys. After extrusion at 260 °C, the maximum tensile strength of Mg-2Sn alloy can reach more than 300 MPa with the addition of 1% Ca and the yield strength can reach 269 MPa, and the reason for the appearance of the high strength is attributed to the large quantity of nano-sized MgSnCa phases and their extremely tiny grain size. Zhao [12] et al. noticed a slight increase in the average grain size after the addition of different contents of Y to Mg-1Sn (at%) alloys. The evolution of the intermetallic compounds in as-extruded alloys was MgSnY→MgSnY + Sn_3_Y_5_→Sn_3_Y_5_. The greatest tensile and yield strengths were obtained with the 3at% Y-added alloy, with somewhat decreased elongation.

For the thermal conductivity of Mg-Sn alloys, a solid solution of Sn in *α*-Mg and the formation of Mg_2_Sn causes lattice defects in the magnesium matrix, which decreases the mean free distance of phonons and electrons, consequently contributing to a decrease in the thermal diffusivity. Moreover, it is known from earlier research that the effect of solid solution strengthening on the thermal conductivity of Mg alloys is substantially more significant than second-phase strengthening [13]. Therefore, some researchers have improved the thermal conductivity of Mg alloys based on adding other elements to consume solid solution atoms that are dissolved in the Mg matrix to generate a new phase. Zhou [14] revealed that the addition of La reduced the amount of solute atoms and eliminated the Sn-enriched region, thus increasing the thermal conductivity of the Mg-2Sn alloys. Kim [15] concluded that adding Sn to Mg-2Ca alloys led to an increase in the MgSnCa phase and a decrease in the Mg_2_Ca phase. Wang [16] suggested that the solute atoms that caused lattice distortion had a greater effect on thermal conductivity compared with the second phases formed in as-cast Mg-Sn-Ca alloys. Zhou [17] found that adding Ca to Mg-4Si alloys had an obvious effect on the Mg_2_Si phase. The highest thermal conductivity was measured when the Ca content reached 0.8 wt.%. Peng [18] showed that as-extruded Mg-2Zn-1Mn-*x*Ce (*x* = 0, 0.2, and 0.6 wt.%) alloys exhibited higher thermal conductivity than the as-cast state due to the precipitation of Mn particles and Ce-containing intermetallic compounds. Liu [19] discovered that with the increase of La content, the volume fraction of Al_11_La_3_ in the Mg-4Al-*x*La (*x* = 2, 4, 6 wt.%) increased because La tends to consume Al to form Al_11_La_3_. Yang [20] revealed that the thermal conductivity of AZ31 and ZK30 alloys could be improved by increasing the Sn content. The thermal conductivities of the ZK30 alloys were obviously higher than those of the AZ31 alloys. This was mainly due to the significantly smaller lattice distortion of the ZK30 alloys compared to that of the AZ31 alloys. Ce is a type of cheap rare-earth element, and its addition to magnesium alloys plays various roles, such as improving the mechanical properties. Özarslan [21] found that adding Ce could refine the grain size of Mg-4Sn alloys, and two new intermetallic compounds (MgSnCe and Ce_5_Sn_4_) were created in the alloys. Yarkada [22] et al. discovered that by adding Ce to Mg-5Sn alloys, efficient grain refinement could be achieved and the production of the new phases, Ce_5_Sn_4_ and CeMgSn, would significantly contribute to the hardness of Mg-5Sn alloys. Moreover, the overall performance of the Ce-added alloy was found to be enhanced compared to the base alloy by examining the mechanical properties under room- and high-temperature conditions. Meanwhile, Kozlov [23] et al. established a thermodynamic database of ternary Mg-Ce-Sn through a combination of the calphad method and laboratory experiments and concluded that MgSnCe is an extremely stable ternary compound. Moreover, the crystal structure and thermodynamic formation of CeMgSn compounds have been thoroughly analyzed by Manfrinetti [24] et al.

In contrast to the investigation of the mechanical properties of Mg-Sn-Ce alloys, no information regarding the thermal conductivity of Mg-Sn-Ce alloys has been reported. To clarify the influence of Ce on Mg-3Sn alloys in as-cast and as-extruded conditions, the microstructure, thermal conductivity, and mechanical properties of as-cast and as-extruded Mg-3Sn alloys with different Ce contents are investigated in this paper.

## 2. Experimental Procedure

Four experimental Mg-3Sn-*x*Ce alloys at various Ce levels (0, 1.0, 3.4, and 4.0 wt.%) were fabricated in this paper. Firstly, magnesium (Wenxiyinguang, Yuncheng, China) with a measured purity of 99.95% was added to a resistance furnace, which was protected by passing a mixture of Ar and SF_6_ gas (Kemeite, Chengdu, China). The preheated pure Sn and Mg-Ce alloys (Baotou Research Institute of Rare Earths, Baotou, China) were then poured into the melt at 680 °C with the designed content. After approximately one hour of refinement at 730 °C, the melt was cast in a metal ingot of specific dimensions. A sampling of the drill chips was carried out on the ingot of the alloy and the actual chemical compositions were obtained, as shown in Table 1.

Subsequently, the as-cast billets were homogenized, and the specific processing was to hold the billets in the muffle furnace at 330 °C over a period of 12 h, then control the heat treatment furnace to raise the temperature to 480 °C at 5 °C/min, and remove it after holding it at 480 °C for 10 h, and subsequently quench them in hot water at 70–80 °C. Cylindrical samples were produced from homogenized ingots with a diameter of 39.6 mm and a length of 40 mm. The samples were extruded at 350 °C with a diameter of 14 mm, extrusion ratio of 8:1, and at a speed of 6 mm/min.

In this experiment, the microstructure of the specimens was observed with a Zeiss Axioscope 5 (Carl Zeiss AG, Jena, Germany) optical microscope and a Zeiss-SIGMA500 (Carl Zeiss AG, Oberkochen, Germany) field emission scanning electron microscope equipped with a Bruker xFlash6160 type energy dispersive spectrometer (EDS). The phase composition was measured using a Rigaku Smart SE X-ray diffractometer. The microstructure and grain orientation of the as-extruded alloys were analyzed using a Nordlys max3 (Oxford instrument, Oxford, Britain) electron backscatter diffractometer (EBSD). Measurements of the tensile properties of the as-cast and as-extruded specimens were taken on an INSTRON-5982 (INSTRON, Norwood, MA, USA) test machine at a velocity setting of 0.5 mm/min.

Thermal diffusivity was measured with a Netzsch LFA 427 (Netzsch, Selb, Germany) instrument at temperatures of 25 °C, 50 °C, 100 °C, 150 °C, and 200 °C. The standard sample size was a small circular plate that was 12.6 mm in diameter and 2.5 mm thick. At least three points were measured at each test temperature to minimize errors. Specifically, the thermal diffusivity of the as-extruded alloys was tested in two directions, one parallel to the extrusion direction and the other perpendicular to the extrusion direction. The ambient density of the sample alloys was achieved by the Archimedes drainage principle by means of a DE-250ME (Hongtu, Guangdong, China) digital density analyzer, and the high-temperature density was calculated according to Equation (1) [25].

(1) $$\rho \left(T\right)=\rho_{0}-0.156\left(T-298\right)$$

where *ρ*(T) is the density at temperature T, *ρ*_0_ is the density at room temperature, and T is the absolute temperature.

The specific heat capacity data for pure Mg, Sn, and Ce at different temperatures can be analyzed using Equations (2)–(4) [26]. The details are as follows:

(2) $$C_{p}\left(\mathrm{Mg}\right)=0.89+4.58\times 10^{-4}T$$

(3) $$C_{p}\left(\mathrm{Sn}\right)=0.18+1.52\times 10^{-4}T$$

(4) $$C_{p}(\mathrm{Ce})=0.16+0.98\times 10^{-4}T$$

where T is the absolute temperature. Therefore, in this paper, the specific heat capacity of ternary Mg-Sn-Ce alloys is given following the Neumann–Kopp rule. Finally, the thermal conductivity of Mg-Sn-Ce alloys with different compositions may be calculated using Equation (5) [27].

(5) $$\lambda =\alpha \cdot \rho \cdot C_{p}$$

where *λ* is the thermal conductivity, *α* is the thermal diffusivity, *ρ* is the density, and *C_p_* is the specific heat capacity.

## 3. Results and Discussion

### 3.1. Microstructure Analysis of As-Cast Alloys

**Figure 1:**
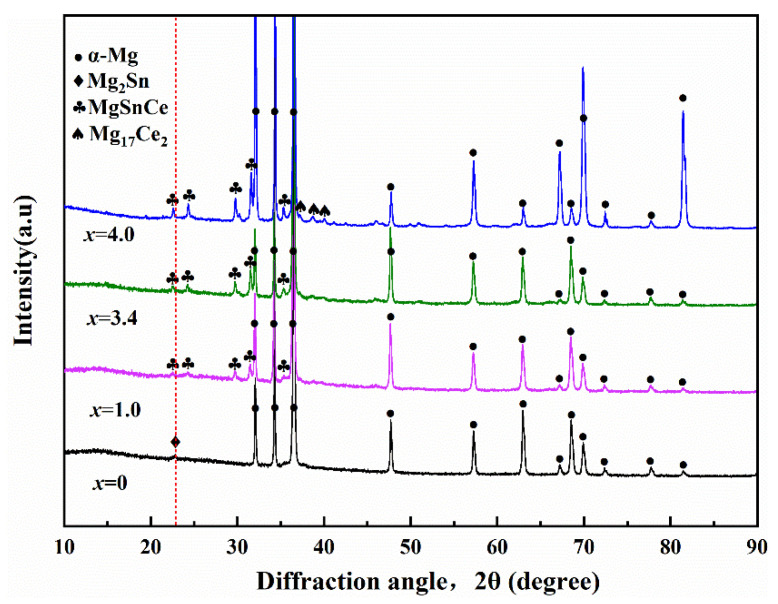
XRD patterns of as-cast Mg-3Sn-*x*Ce alloys.

X-ray diffraction patterns of as-cast Mg-3Sn-*x*Ce (*x* = 0, 1.0, 3.4 and 4.0 wt.%) alloys are presented in Figure 1.

It can be seen that the Mg-3Sn alloy is mainly composed of *α*-Mg and Mg_2_Sn phases; owing to the large solid solubility of Sn in *α*-Mg, only a weak peak of Mg_2_Sn is observed at 22.7°. Adding Ce creates some new diffraction peaks, which implies the creation of some new second phases, and the Mg_2_Sn peak was not detected. It can be observed that the diffraction intensity of the newly formed MgSnCe phase rises gradually as the Ce content is increased. Furthermore, the Mg_17_Ce_2_ phases were detected in the Mg-3Sn-4Ce alloy.

**Figure 2:**
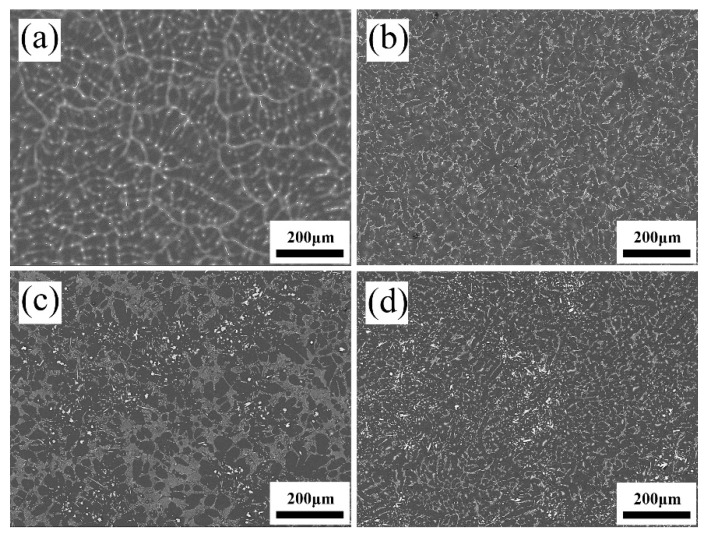
SEM images of as-cast Mg-3Sn-*x*Ce alloys. (**a**) *x* = 0, (**b**) *x* = 1.0, (**c**) *x* = 3.4, (**d**) *x* = 4.0.

**Figure 3:**
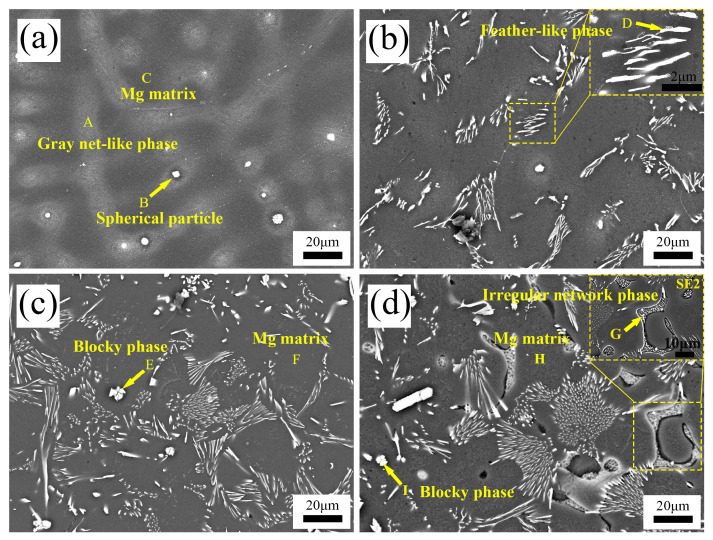
SEM microstructures of as-cast Mg-3Sn-*x*Ce alloys. (**a**) *x* = 0, (**b**) *x* = 1.0, (**c**) *x* = 3.4, (**d**) *x* = 4.0.

Figure 2 and Figure 3 illustrate the lower and higher magnification SEM patterns of as-cast Mg-3Sn alloys with different levels of Ce added, respectively.

It is evident that the mean grain size in the Mg-3Sn alloy decreases as the Ce content increases, as indicated in Figure 2. The grain size was counted by Image-Pro Plus 6.0 software and at least 50 grains were captured for each SEM image. Based on the grain size calculation, the influence of adding Ce on the grain size of the alloys is listed in Table 2. This demonstrates the effectiveness of Ce in reducing the grain size of the Mg-3Sn alloys.

As shown in Figure 3a, some light gray net-like phases (point A) and spherical particles (point B) can be observed enriched at the grain boundaries. According to the atomic ratio of Mg to Sn in Table 3, it can be determined that the light gray net-like phases are the α-Mg and Mg_2_Sn continuous eutectic structure, and spherical particles are the Mg_2_Sn phases. The Mg matrix was also analyzed by EDS (point C), and the point C results suggest that some Sn (2.43 at.%) dissolved into α-Mg grains.

Moreover, a slight quantity of Mg_2_Sn exists in a dissociated eutectic form as spherical particles. As the Ce concentration increases to 1.0 wt.%, the microstructure of the alloys exhibits a significant evolution, and most of the second-phase morphology transforms into feather-like (point D), as depicted in Figure 3b. Moreover, combined with the EDS analysis of points D, which consists of 89.41 at.% Mg, 5.44 at.% Sn, and 5.15 at.% Ce, the atomic ratios of Sn and Ce are 1.05, and thus the second phase is the MgSnCe phase. This is in agreement with the research of Özarslan [21] and Yarkada [22].

As the Ce content continues to increase to 3.4 wt.%, as shown in Figure 3c, the previously clearly identified grain boundaries almost disappeared, and a large number of intermetallic compounds in *α*-Mg could be detected dispersed as tiny particles, and some new phases (point E) appeared, showing irregular blocks and rods, combined with the analysis of E-points, which consist of 58.72 at.% Mg, 21.23 at.% Sn, and 20.05 at.% Ce. The ratio of Sn atoms to Ce atoms is almost 1:1 and the formed blocky second phase is MgSnCe. Some irregular phases (point G) occur in the Mg-3Sn-4.0Ce alloy and display a network distribution, as shown in Figure 3d, combined with the analysis of the G-points, which consists of 95.53 at.% Mg and 4.47 at.% Ce, indicating that a new Mg_17_Ce_2_ phase is formed in the matrix. Similarly, the Mg matrix in Figure 3c,d was analyzed by EDS and the Sn content at point F and point H were 0.15 at.% and 0.07 at.%, respectively. We see that the Sn atoms solidly dissolved in the Mg matrix are decreasing in number in comparison with Figure 3a.

Hence, it can be concluded that as the Ce levels rise between 0 and 4.0 wt.%, the composition and morphology of the second phase in the Mg-3Sn alloys evolves as Mg_2_Sn (larger spherical particles)→MgSnCe (feather-like)→MgSnCe (irregular blocks and rods)→MgSnCe (irregular blocks and rods) + Mg_17_Ce_2_ (irregular network).

**Figure 4:**
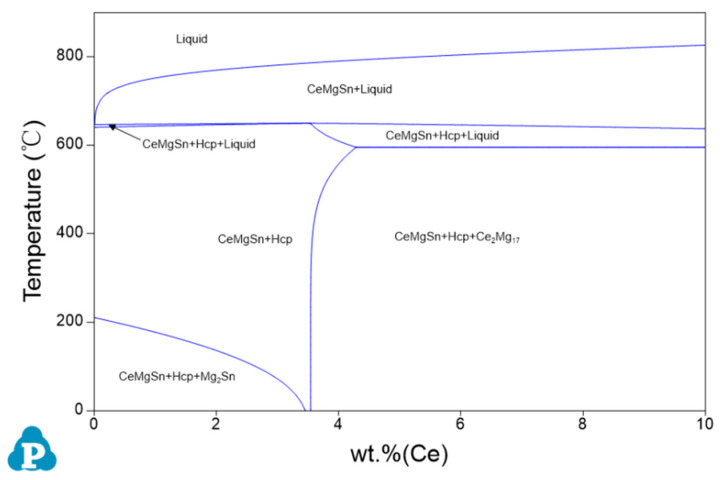
Phase diagram of the Mg-3Sn-*x*Ce system alloy with Mg-rich angular vertical interface.

Figure 4 presents the thermodynamic Pandat software-calculated Mg-rich corner phase diagrams of the Mg-3Sn-*x*Ce system alloys. In the case of the Mg-3Sn-4.0Ce alloy, during the initial solidification stage, the feathery CeMgSn phase forms while crossing the L + CeMgSn region, and then undergoes pseudo-binary eutectic (CeMgSn + L→CeMgSn + *α*-Mg) and ternary eutectic (L→CeMgSn + *α*-Mg + Mg_17_Ce_2_) reactions, respectively, which ultimately lead to the formation of the irregular blocky and rod-like CeMgSn and irregular network Mg_17_Ce_2_ phases.

**Figure 5:**
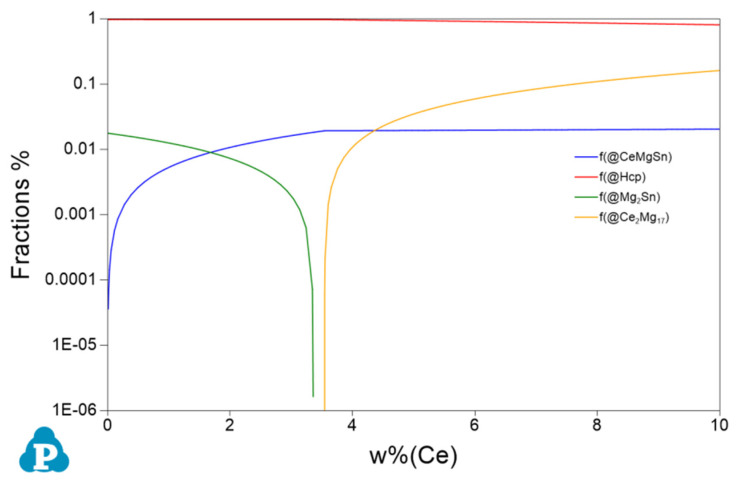
Variation of intermetallic compounds with Ce content of Mg-3Sn-*x*Ce alloy at 25 °C.

Figure 5 shows the changes in phase compositions with increasing Ce content. With a Ce level below 3.3 wt.%, Mg_2_Sn and CeMgSn are formed. With increasing amounts of Ce, the fraction of CeMgSn increases, while the concentration of Mg_2_Sn decreases from 1.79 wt.% to zero. It suggests that the phase composition is *α*-Mg, Mg_2_Sn, and CeMgSn in the Mg-3Sn-1.0Ce alloy. The new phase, Mg_17_Ce_2_, is formed while the Ce content continues to rise from 3.3 wt.% until it reaches 3.5 wt.%. Combined with the phase diagram of Figure 4, the only phases in the Mg-3Sn-3.4Ce alloy from the liquid state to the solidification finish are *α*-Mg and CeMgSn, whereas the phases are composed of *α*-Mg, Mg_17_Ce_2_, and CeMgSn in the Mg-3Sn-4.0Ce alloy. These results are in agreement with those analyzed in Figure 3.

**Figure 6:**
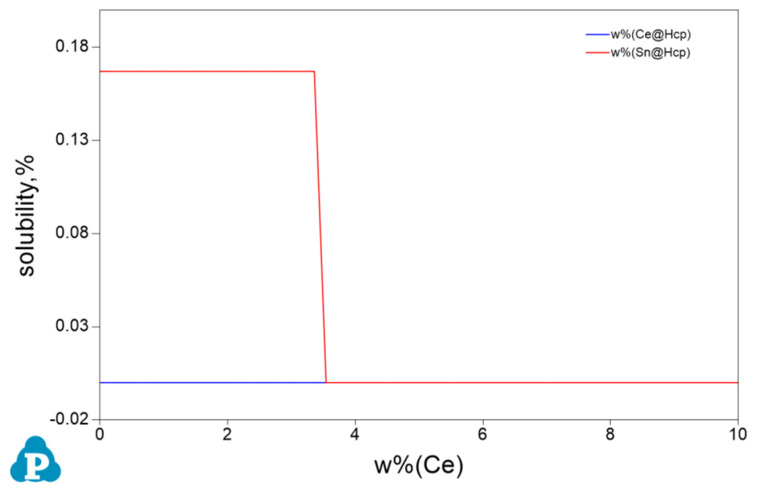
Variation of dissolved Ce and Sn concentration with Ce content in *α*-Mg matrix of Mg-3Sn-*x*Ce alloy at 25 °C.

The variation of solubility of the Sn and Ce elements in α-Mg with a Ce content is displayed in Figure 6.

It can be noted that the solubility of Sn content in α-Mg is steady at a minor addition of Ce, and the solubility of Sn in *α*-Mg decreases drastically from 16.7% to zero when the addition of Ce exceeds 3.3 wt.%. This is because the addition of Ce to Mg-3Sn consumes the Sn atoms solidly dissolved in α-Mg and prefers to form the CeMgSn phase.

### 3.2. Microstructure and Textures of As-Extruded Alloys

**Figure 7:**
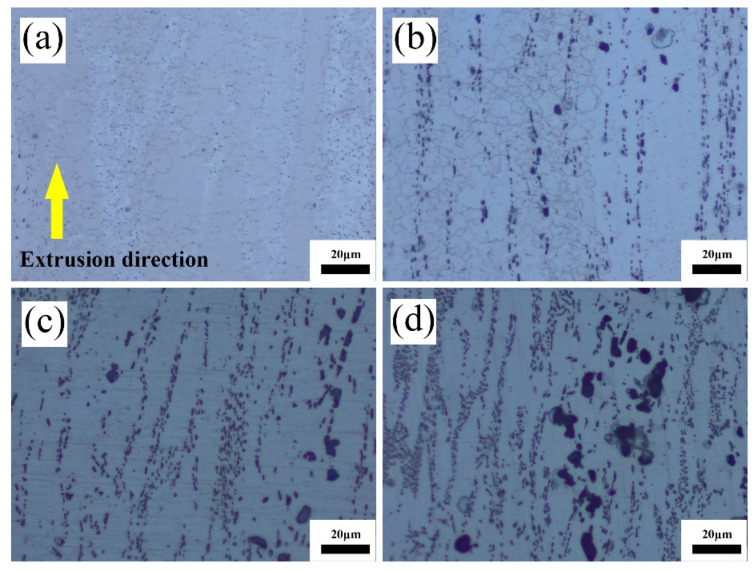
Optical micrographs of as-extruded Mg-3Sn-*x*Ce alloys. (**a**) *x* = 0, (**b**) *x* = 1.0, (**c**) *x* = 3.4, (**d**) *x* = 4.0.

Figure 7 presents some optical micrographs of the Mg-3Sn-*x*Ce alloys after extrusion under various Ce influences.

After extrusion, for the Ce-free Mg alloy, there are no visible second-phase precipitates, and the matrix consists of fine equiaxed grains and undynamically recrystallized (unDRX) grains elongated along the ED direction. The other three alloys with added Ce present obvious second-phase precipitates, and the quantities of these precipitates gradually rise along the direction of extrusion as the Ce concentration grows, similar to the study by Hao [28] et al. The addition of the Ce content exceeding 3.4 wt.% is accompanied by the generation of larger blocky second-phase precipitates.

**Figure 8:**
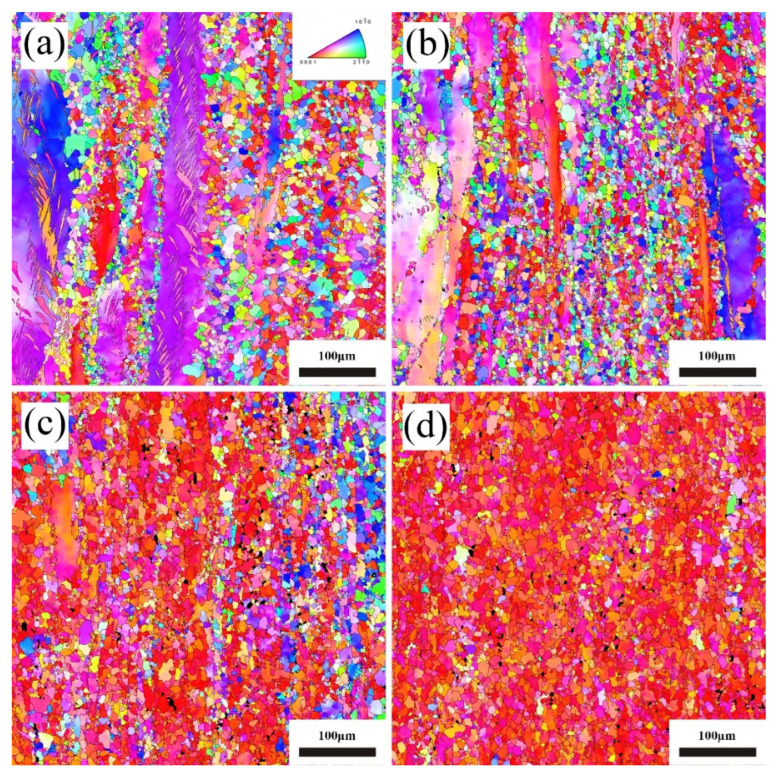
EBSD inverse pole figure maps of as-extruded Mg-3Sn-*x*Ce alloys. (**a**) *x* = 0, (**b**) *x* = 1.0, (**c**) *x* = 3.4, (**d**) *x* = 4.0.

To explore to influence of Ce on the microstructural evolution of Mg-Sn alloys during plastic deformation, EBSD tests were carried out on the extruded samples. Figure 8 illustrates EBSD inverse pole figure maps of as-extruded Mg-3Sn-*x*Ce alloys with various loadings of Ce.

The presence of twins in as-extruded the Mg-3Sn alloy is visible, corresponding to the results of the optical micrographs (OM) in Figure 7a. This may be due to the insufficient driving force for recrystallization caused by the lack of a deformation temperature. Figure 8a–d demonstrates that there is a considerable quantity of deformed grains that are elongated, leading to the generation of a significant amount of deformation textures, which cause a high density of dislocations to accumulate here.

**Figure 9:**
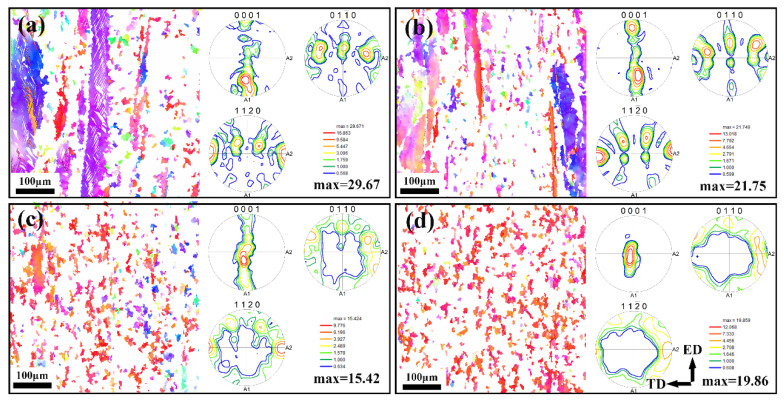
EBSD inverse pole figure maps and pole figures of uncrystallized structure of as-extruded Mg-3Sn-*x*Ce alloys. (**a**) *x* = 0, (**b**) *x* = 1.0, (**c**) *x* = 3.4, (**d**) *x* = 4.0. (ED: extrusion direction, TD: transverse directionl direction).

The texture of these deformation grains is analyzed as shown in Figure 9.

It can be seen that as the Ce concentrations rise, the texture of these deformed grains decreases from a maximum pole density of 29.67 mrd (Mg-3Sn alloy) to 15.42 mrd (Mg-3Sn-3.4Ce alloy), and then increases to 19.86 mrd (Mg-3Sn-4.0Ce alloy), as shown in Figure 9a–d. This is because the addition of Ce weakens the texture of the uncrystallized grains and increases the number of second-phase particles in the alloy, which impedes the dislocation movement during deformation, increases the deformation energy storage, and results in an increment of the recrystallization driving force.

**Figure 10:**
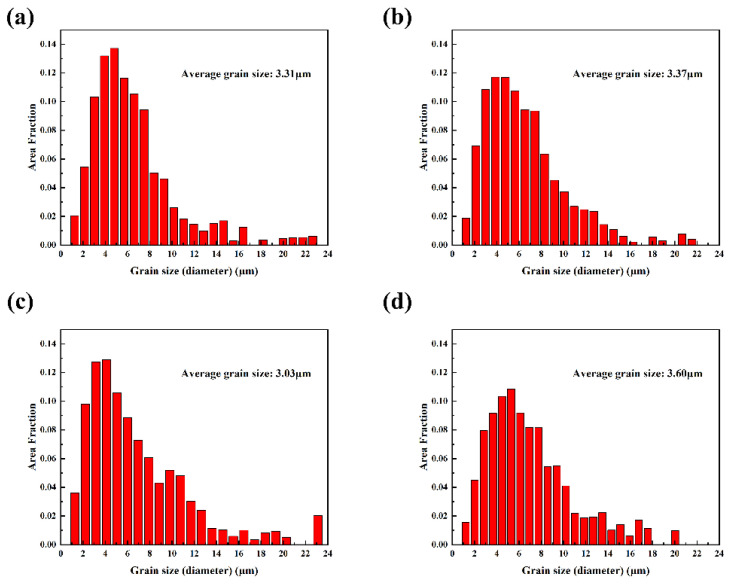
Grain size (in the recrystallised area) distribution of as-extruded Mg-3Sn-*x*Ce alloys (**a**) *x* = 0, (**b**) *x* = 1.0, (**c**) *x* = 3.4, (**d**) *x* = 4.0.

The grain size was measured and counted for the recrystallized areas in Figure 8, as shown in Figure 10. The size of the totally recrystallized grains was calculated to be about 3 μm. Comparing Table 2 and Figure 10, it is evident that after extrusion the average grain size is markedly improved.

### 3.3. Thermal Conductivity of As-Cast and As-Extruded Alloys

**Figure 11:**
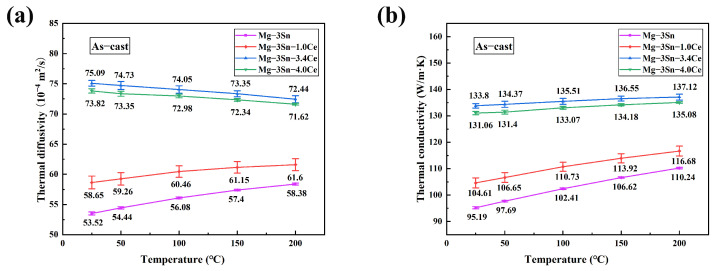
(**a**) Thermal diffusivity and (**b**) thermal conductivity of as-cast Mg-3Sn-*x*Ce alloys at different temperatures.

Figure 11 shows the thermal diffusivity and thermal conductivity of as-cast Mg-3Sn-*x*Ce alloys with various concentrations of Ce at different temperatures. We see that the thermal conductivity of the as-cast alloy improves as the Ce percentage grows, and achieves a peak at a Ce content of 3.4 wt.%.

The thermal conductivity increased from 95.19 W/(m·K) to 133.8 W/(m·K), which is a 40.6% improvement in thermal conductivity, whereas adding excessive amounts of elemental Ce (*x* = 4.0) reduces the thermal conductivity. Moreover, it is obvious that the thermal conductivity of as-cast alloys is higher at elevated temperatures, which is in agreement with the studies of Yang [29] and Yamasaki [30] et al. It is attributed to the influence of temperature, where the Sn atoms solidly dissolved in the Mg matrix form intermetallic compounds, which reduces the extent of the lattice defects. Meanwhile, as the temperature rises, the lattice expands and the speed of electron and phonon movement is accelerated. This is beneficial for the transport of thermal energy by electrons and phonons, thus improving thermal conductivity.

Depending on the mechanism of thermal conduction, free electrons determine the efficiency of thermal transfer in metals. The most considerable influence on the thermal conductivity of a lot of non-ferrous alloys is the lattice distortion due to solid-solution atoms and increased interface with the intermediate compounds. Both lattice distortions and phase interfaces become sources of scattering of phonons and free electrons, resulting in reduced thermal diffusivity and thermal conductivity [31]. Since the maximum solubility of Sn in magnesium can reach 14.5 wt.% [32], a significant number of Sn atoms would be solute in the matrix of Mg and a minor quantity of phases of Mg_2_Sn would be generated around the grain boundaries in the as-cast Mg-3Sn alloy. Due to lattice distortion caused by the Sn atoms, the thermal conductivity of the Mg-3Sn alloy is only 95.19 W/(m·K), significantly below that of 158 W/(m·K) for Mg. The addition of Ce forms a new MgSnCe phase, depletes the Sn that is solidly dissolved into the matrix of Mg, and reduces the *α*-Mg + Mg_2_Sn eutectic mix. The results of point C, point F, and point H from the EDS in Table 3 illustrate that the Sn solid-solution levels in the Mg-3Sn alloy matrix reduce from 8.63 wt.% to 0.74 wt.% and 0.35 wt.%, containing 3.4 wt.% and 4 wt.% of added Ce, respectively.

The impact of second-phase strengthening on thermal conductivity is considerably weaker than that of the solid solution strengthening in the Mg-3Sn alloy. Consequently, despite the fact that the addition of Ce results in the creation of a new second phase and a new phase interface, the thermal conductivity of the Mg-3Sn alloy remains increased. However, the results of point G in Table 3 show that a minor quantity of Ce is dissolved in the *α*-Mg matrix at 1.64 wt.% when 4.0 wt.% Ce is added. In addition, a new Mg_17_Ce_2_ phase is formed. Thus, under the influence of these two aspects, the thermal conductivity of the Mg-3Sn-4Ce alloy decreases slightly in comparison with Mg-3Sn-3.4Ce alloy.

**Figure 12:**
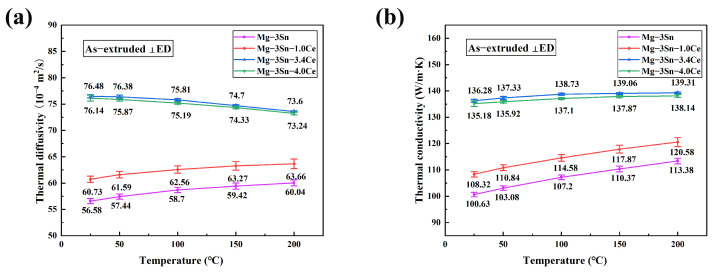
(**a**) Thermal diffusivity and (**b**) thermal conductivity of as-extruded (perpendicular to ED) Mg-3Sn-*x*Ce alloys at different temperatures.

**Figure 13:**
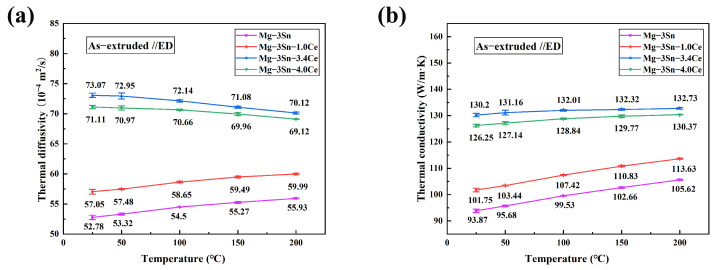
(**a**) Thermal diffusivity and (**b**) thermal conductivity of as-extruded (parallel to ED) Mg-3Sn-*x*Ce alloys at different temperatures.

Figure 12 and Figure 13 show the anisotropy of the thermal diffusivity and thermal conductivity of the as-extruded Mg-3Sn alloys with various Ce contents.

It is obvious that the thermal conductivity parallel to the extrusion direction (ED) is lower than that perpendicular to the ED. Similar to the as-cast alloys, the thermal conductivity of as-extruded alloys rises with Ce levels, reaching a maximum level at a Ce content of 3.4%. The thermal conductivity parallel to the ED and perpendicular to the ED increased from 93.87 W/(m·K) and 100.63 W/(m·K) to 130.20 W/(m·K) and 136.28 W/(m·K), which represents an approximate growth of 38.7% and 35.4% in comparison. Like the as-cast alloys, the thermal conductivity of the as-extruded alloys increases as the temperature elevates, whereas the thermal conductivity of Mg-3Sn-3.4Ce can reach 139.31 W/(m·K) perpendicular to the ED at 473 K.

As can be seen by comparison with Figure 11, after extrusion deformation, the thermal conductivity parallel to the ED slightly decreases compared to the as-cast alloy, while the thermal conductivity perpendicular to the ED slightly increases. This is in agreement with the studies of Yuan [33] et al. The grain size of the Mg-3Sn-*x*Ce alloys decreases significantly after extrusion, leading to an enhancement of the scattering of free electrons. However, on the one hand, it causes a new second phase to occur consisting of Sn atoms solidly dissolved in *α*-Mg matrix, which promotes thermal conductivity; on the other hand, texture formation by the extrusion process is one of the most important elements that affects thermal conductivity. As shown in Figure 8a–d, a huge amount of uncrystallized deformed grains are present in the Mg-3Sn-xCe alloys, which are found to be distributed in the strongest position of the polar density in the polar diagram, thus it is necessary to focus on the texture analysis of the uncrystallized deformed grains. There is clearly a strong texture in the uncrystallized deformed grains as illustrated by Figure 9, and the texture strength decreases firstly as the proportion of Ce grows from 29.67 mrd (Mg-3Sn) to 15.42 mrd (Mg-3Sn-3.4Ce), and the addition of a moderate amount of Ce weakened the texture of the uncrystallized deformed grains, thus improving the thermal conductivity. In contrast, adding excessive Ce (4 wt.%) strengthens the texture of the deformed grains, resulting in a minor reduction in thermal conductivity. Therefore, regarding the Mg-Sn-Ce alloys with a great deal of uncrystallized deformed grains, the strength of the deformed grain texture has a more substantial effect on the thermal conductivity, and weakening the deformed grain texture helps to improve thermal conductivity.

### 3.4. Mechanical Properties of As-Cast and As-Extruded Alloys

**Figure 14:**
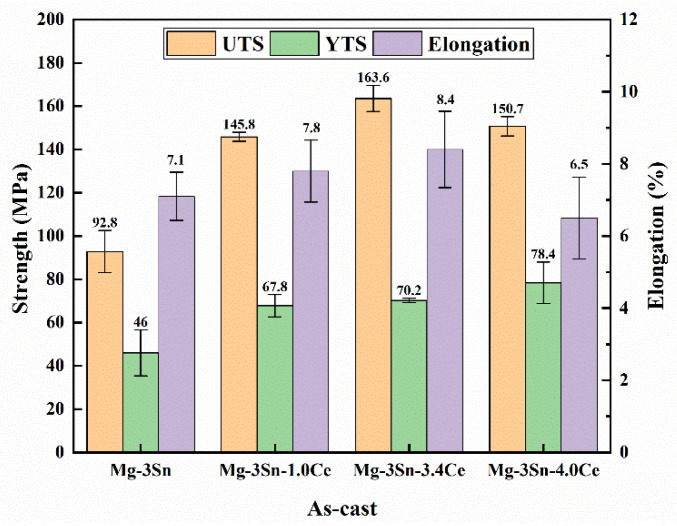
Mechanical properties of as-cast Mg-3Sn-*x*Ce alloys.

**Figure 15:**
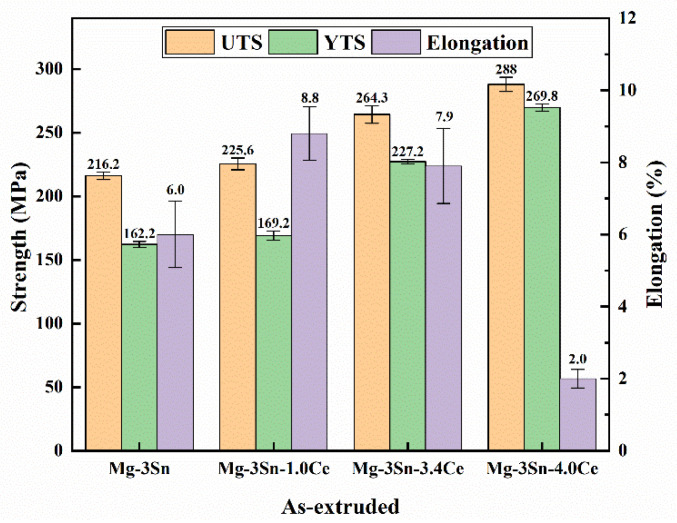
Mechanical properties of as-extruded Mg-3Sn-*x*Ce alloys.

The mechanical properties of Mg-3Sn-*x*Ce (*x* = 0, 1, 3.4, and 4 wt.%) in the as-cast and as-extruded state are illustrated in Figure 14 and Figure 15.

The yield strength of the Mg-3Sn-*x*Ce alloy improves as the Ce concentration is added under the as-cast state. This is attributed to the effective grain refinement through the addition of Ce, as shown in Table 2. Another aspect is that the addition of Ce introduces a considerable amount of second phase CeMgSn, which can inhibit the dislocation movement effectively. However, when the Ce content is up to 4%, the elongation decreases clearly, which is associated with producing brittle intermetallic compounds and Mg_17_Ce_2_ phases, and these brittle intermetallic compounds can cause stress concentration in tensile deformation, where cracks emerge and expansion takes place. After extrusion, the reason for the improved mechanical properties of the three alloys is mainly because of obvious dynamic recrystallization, grain refinement, and a large number of dislocation build-ups that occurred during the hot extrusion process. Precipitation particles of intermetallic compounds emerge clearly, which are dispersed parallel to the ED, and the volume fraction gradually rises with the content of Ce, which contributes to enhancing the yield strength of these alloys. When 4 wt.% of Ce is added, according to Figure 7, more precipitated particles with a larger morphology are present in the alloy, which are prone to producing stress concentrations around the interface, and thus the elongation of the Mg-3Sn-4.0Ce alloy is lower. Overall, it is observed that the tensile and yield strengths of the as-extruded Mg-3Sn-3.4Ce alloy can reach 264.3 and 227.2 MPa, respectively. Furthermore, the thermal conductivity perpendicular to the ED at room temperature is 136.28 W/(m·K), which can be used as a thermally conductive device with a lower cost and a more superior thermal conductivity and comprehensive mechanical properties.

## 4. Conclusions

The microstructural evolution, improvement of mechanical properties, and thermal conductivity of Mg-3Sn alloys with various levels of Ce concentrations were researched and the findings are presented below:

1. A new second MgSnCe phase appeared after adding Ce to the as-cast Mg-3Sn alloy, and the Mg_17_Ce_2_ phase emerged as the Ce content increased to 4%.
2. The addition of Ce reduced the number of solute atoms of Sn, resulting in a significant improvement in the thermal conductivity of as-cast Mg-3Sn alloys. However, an excess of Ce causes a formation of the Mg_17_Ce_2_ phase, which has a negative effect on the thermal conductivity.
3. After extrusion, the thermal conductivity is improved, owing to the added Ce, which weakens the texture of the deformed grains.
4. The addition of Ce increased the yield strength of the as-cast alloy. This is because Ce could refine the grains, as well as form the second phase MgSnCe to hinder the dislocation movement. The properties are further enhanced after extrusion due to the recrystallization process.

## Figures and Tables

**Table 1:** Actual chemical composition of as-cast Mg-3Sn-*x*Ce (*x* = 0, 1.0, 3.4, and 4.0 wt.%) alloys.

Alloys	Composition (wt.%)		
	Sn	Ce	Mg
*x* = 0	2.91	-	Bal.
*x* = 1.0	2.90	1.22	Bal.
*x* = 3.4	3.34	3.33	Bal.
*x* = 4.0	3.10	3.90	Bal.

**Table 2:** The effect of Ce addition on grain size of Mg-3Sn alloys.

Alloys	Grain Size (μm)
Mg-3Sn	25.99 ± 4.79
Mg-3Sn-1.0Ce	17.37 ± 3.59
Mg-3Sn-3.4Ce	15.70 ± 3.63
Mg-3Sn-4.0Ce	14.39 ± 3.35

**Table 3:** EDS results of the corresponding points in Figure 3.

Points	Mg		Sn		Ce	
	wt.%	at.%	wt.%	at.%	wt.%	at.%
A	89.17	97.57	10.83	2.43	-	-
B	30.73	68.42	69.27	31.58	-	-
C	91.37	98.10	8.63	1.90	-	-
D	61.38	89.41	18.25	5.44	20.37	5.15
E	21.12	58.72	37.30	21.23	41.58	20.05
F	97.90	99.61	0.74	0.15	1.36	0.24
G	78.76	95.53	-	-	21.24	4.47
H	98.02	99.64	0.35	0.07	1.64	0.29
I	43.30	80.25	26.30	9.98	30.39	9.77

## Data Availability

The raw/processed data required to reproduce these findings cannot be shared at this time because the data also form part of an ongoing study.
